# A systematic review on the role of glucagon-like peptide-1 receptor agonists on alcohol-related behaviors: potential therapeutic strategy for alcohol use disorder

**DOI:** 10.1017/neu.2025.6

**Published:** 2025-02-19

**Authors:** Yang Jing Zheng, Crystaleene Soegiharto, Hezekiah C.T. Au, Kyle Valentino, Gia Han Le, Sabrina Wong, Kayla M. Teopiz, Taeho Greg Rhee, Hernan F. Guillen-Burgos, Bing Cao, Roger S. McIntyre

**Affiliations:** 1 Brain and Cognition Discovery Foundation, Toronto, ON, Canada; 2 Mood Disorder Psychopharmacology Unit, University Health Network, Toronto, ON, Canada; 3 Institute of Medical Science, University of Toronto, Toronto, ON, Canada; 4 Department of Pharmacology and Toxicology, University of Toronto, Toronto, ON, Canada; 5 Department of Psychiatry, University of Toronto, ON, Canada; 6 Department of Psychiatry, Yale School of Medicine, New Haven, CT, USA; 7 Department of Public Health Sciences, University of Connecticut School of Medicine, Farmington, CT, USA; 8 Universidad El Bosque, Faculty of Medicine, Center for Clinical and Translational Research, Bogota, DC, Colombia; 9 Universidad Simon Bolivar, Center for Clinical and Translational Research, Barranquilla, Colombia; 10 Pontificia Universidad Javeriana, Department of Psychiatry and Mental Health, Hospital Universitario San Ignacio, Bogota, DC, Colombia; 11 Key Laboratory of Cognition and Personality, Faculty of Psychology, Ministry of Education, Southwest University, P. R. China

**Keywords:** Exenatide, Liraglutide, Dulaglutide, Semaglutide, GLP-1, Alcohol use disorder, Alcohol consumption, Depression, Bipolar disorder

## Abstract

**Introduction:**

Extant literature implicates the role of glucagon-like peptide-1 (GLP-1) and GLP-1 receptor agonists (GLP-1RAs) on modulating alcohol-associated behaviours, with a particular emphasis of these agents on neural circuits subserving reward and appetite control. Herein, we explore the potential effects of GLP-1RAs on alcohol-associated behaviours in brain regions implicated in reward processing facilitating the repurposing of these agents for the treatment and prevention of problematic drinking. Understanding how GLP-1’s analogues interact with alcohol-related behaviours may underscore the development of therapeutic strategies for alcohol use disorder (AUD) and those with comorbid metabolic disorders.

**Methods:**

A systematic review was conducted, wherein relevant literature was identified through Web of Science, PubMed, and OVID (MedLINE, Embase, AMED, PsycInfo, JBI EBP) from database inception to October 27th, 2024. Preclinical and clinical studies examining the association between GLP-1RAs and alcohol-related behaviours were assessed.

**Results:**

Preclinical studies (*n* = 19) indicate that GLP-1RAs attenuate alcohol-related behaviours, with exenatide demonstrating significant dose-dependent effects in high alcohol-consuming phenotypes. Semaglutide and liraglutide are associated with reduced alcohol intake, though their effects were often transient. In human studies (*n* = 2) with AUD, semaglutide significantly reduced alcohol consumption, while exenatide showed mixed results, with reductions in alcohol drinking within high BMI subpopulations.

**Discussion:**

Extant preclinical and clinical literature provides preliminary support for the potential therapeutic role of GLP-1RAs in attenuating alcohol consumption and preference. There is a need for large well controlled studies evaluating the effect of GLP-1RAs as a treatment strategy for behavioural modifications in individuals living with alcohol use disorder.

Summations

- Glucagon-like peptide-1 receptor agonists (i.e., exenatide, liraglutide, and semaglutide) result in attenuation of alcohol-related behaviours, including alcohol consumption, alcohol preference, and alcohol-induced neurochemical responses in animal models.
- Effects of glucagon-like peptide-1 receptor agonists vary with specific brain regions targeting in animal models, with observed effectiveness in the NAc and VTA implicated in alcohol use disorder.
- Results on whether glucagon-like peptide-1 receptor agonists are able to reduce compulsive drinking behaviour in individual’s living with alcohol use disorder are mixed, and there remains a need for adequate and well studies.

Considerations

- There are limited clinical studies assessed within this review, with contrasting findings on the efficacy of glucagon-like peptide-1 receptor agonists on alcohol usage.
- The transient nature of glucagon-like peptide-1 receptor agonists were not assessed in the literature reviewed in this paper. Notably, receptor desensitisation and neuroadaptations may limit the efficacy of the analogues.
- Although treatment combinations have been suggested to yield greater efficacy, the reviewed literature did not examine the synergistic effects of varied glucagon-like peptide-1 receptor agonists on alcohol-related behaviours.

## Introduction

Glucagon-like peptide-1 (GLP-1) has been widely used to treat type 2 diabetes mellitus (T2 DM), with extant literature supporting its role in glycemic and appetite control (Andersen *et al*., 2018). This peptide consists of 30 amino acids and is synthesised in the endocrine epithelial L cells, pancreatic alpha cells, and by a subset of neurons in the nucleus of the solitary tract (NTS) in the brainstem (Andersen *et al*., 2018; Holst, 2024; Zheng *et al*., 2024). Stimulated by ingested macronutrients, GLP-1 is secreted postprandial in response to an oral glucose load (Holst, 2024). Enteroendocrine L cells, responsible for secreting GLP-1, are distributed throughout the gastrointestinal tract, with proximal L cells playing a crucial role in elevating GLP-1 levels in plasma (Holst, 2024; Zheng *et al*., 2024).

GLP-1 has only one known receptor, GLP-1 receptor (GLP-1R), which is widely expressed throughout the body, including the digestive system, cardiovascular system, central nervous system (CNS), and peripheral nervous system (Holst, 2024; Au *et al*., 2024). Notably, GLP-1Rs in circumventricular organs are targets for peripheral GLP-1, particularly after large meals or under conditions like rapid gastric emptying (Holst, 2024). Within the CNS, these receptors are predominantly expressed in brain regions involved in processing rewards, including the ventral tegmental area (VTA) and nucleus accumbens (NAc) (Marty *et al*., 2020). Recent literature demonstrated a link between GLP-1R signalling and dopamine (DA) mechanisms within brain regions involved in substance usage and addiction (Jensen *et al*., 2020; Au *et al*., 2024). Particularly, GLP-1R activation and DA regulation may underlie GLP-1R’s effects on reward, provided dopamine transporter’s (DAT) role in regulating DA neurotransmission. As these regions are implicated in understanding addiction and substance use, as they regulate rewarding effects of substance use, GLP-1Rs may have implications for modulating behaviours associated with appetite and alcohol consumption (Marty *et al.*, 2020; Badulescu *et al.*, 2024).

As an agonist to GLP-1 (GLP-1RAs), GLP-1RAs can serve as a mediator of aforementioned reward and appetite associated signalling. GLP-1RAs, including semaglutide, liraglutide, tirzepatide, and exenatide, are drugs approved by the US Food and Drug Administration for the treatment of obesity and T2 DM (Thomsen *et al*., 2018; Holst, 2024). Regarding its underlying mechanisms, these agonists activate GLP-1R, mimicking the effects of endogenous GLP-1, and resulting in enhanced insulin secretion, attenuated glucagon release, reduced gastric emptying rate, and suppressed central appetite (Zheng *et al*., 2024). Similarly to GLP-1 and its analogues, GLP-1RAs can modulate food intake and hedonic eating by targeting neural substrates, particularly within the mesolimbic reward pathways (e.g., VTA and NAc), altering satiety and reward responses to salient substances often subjected to abuse (Eren-Yazicioglu *et al*., 2021; Zheng *et al*., 2024). GLP-1RAs have further been demonstrated to regulate DA signalling through a process tightly controlled by the DAT, implicating its role in regulating the reward pathway (Jensen *et al*., 2020). Notwithstanding, GLP-1RAs effects on substance-related reward behaviours and GLP-1RAs’ potential to modulate alcohol consumption remains underexplored. The mesolimbic pathways targeted by GLP-1RAs play an integral role in the rewarding effects of alcohol consumption, influencing alcohol seeking motivation and reinforcement. By modulating these pathways, GLP-1RAs serve as a potential therapeutic mechanism to reduce alcohol intake via alteration of reward process pathways. Despite this, the role of GLP-1RAs in alcohol use disorder (AUD) remains largely underexplored, despite their established efficacy in managing metabolic disorders. Alcohol consumption is widespread globally, and its potential benefits and risks have sparked considerable debate among researchers. It is associated with all-cause, cardiovascular disease, and cancer mortality, among other conditions (Tian *et al*., 2023). AUD is a chronic, relapsing condition marked by an irresponsible compulsion to habitually seek alcohol, inability to control alcohol intake, and the development of a negative emotional states leading to dependence on alcohol and associated adverse health concerns (Marty *et al*., 2020). Alcohol’s debilitating effects can further extend to unintentional injury and suicide, contributing to pressing consequences for the engaged individual, relatives, and society at large (Klausen *et al*., 2022). Although the FDA has approved several agents for the treatment of AUD – including disulfiram, naltrexone, and acamprosate – many individuals do not adequately benefit from these treatments (Litten *et al*., 2016). With AUD remaining a global leading cause of preventable morbidity and mortality, understanding GLP-1 RA’s potential in regulating alcohol consumption could open new avenues for therapeutic approaches. As such, this review strives to investigate this potential therapeutic avenue, addressing the significant gap in the development of effective treatment strategies for AUD. Assessment of literature aims to provide an overview of current trends in GLP-1RAs administration in modulation of AUD and related behaviour.

## Methods

### Search strategy

This systematic review was conducted in adherence to the 2020 Preferred Reporting Items for Systematic Reviews and Meta-Analyses (Page *et al*., 2021). Relevant literature was extracted from the following databases: Web of Science, OVID (MedLINE, Embase, AMED, PsychInfo, JBI EBP), and PubMed were used to systematically search for relevant articles from database inception to October 27th, 2024. The search queries used for this review is of the following: (‘GLP-1’ OR ‘Glucagon-Like Peptide-1’ OR ‘Glucagon-Like Peptide 1’ OR ‘GLP-1 Agonist’ OR ‘Glucagon-Like Peptide-1 Agonist’ OR ‘Glucagon-Like Peptide 1 Agonist’ OR ‘Semaglutide’ OR ‘Dulaglutide’ OR ‘Trulicity’ OR ‘Exenatide’ OR ‘Liraglutide’ OR ‘Lixisenatide’ OR ‘Tirzepatide’) **AND** (‘Alcohol’ OR ‘Alcohol use disorder’ OR ‘AUD’ OR ‘Alcoholism’ OR ‘Ethanol Administration’ OR ‘Ethanol’).

### Study selection and inclusion criteria

Articles obtained from databases were screened through Covidence, wherein duplicated articles were automatically excluded (Systematic review management, 2023). Two independent reviewers (H.A. and Y.J.Z.) reviewed the titles and abstracts according to the inclusion and exclusion guidelines (Table 1). Furthermore, relevant English-language primary and secondary sources were then examined through full-text screening and included for extraction if they reported on GLP-1RA-associated changes to alcohol or ethanol consumption. Discrepancies were resolved following thorough discussion between reviewers.

**Table 1.** Eligibility criteria

Inclusion Criteria	A primary or secondary study, Preclinical or clinical studies, Mentions intervention or observational studies, Includes usage of GLP-1 and GLP-1RAs, Measurement of alcohol-related activities, Full-text article available online, English language.
Exclusion Criteria	Non-primary or secondary research (i.e., literature reviews, systematic reviews, meta-analyses, posters, abstracts, guidelines, protocols and theses), No measurement of alcohol-related reward processing, Reports an association without statistics, Full-Text is not available.

### Data extraction

Literature applicable to alcohol-associated behaviours and GLP-1RA administration were obtained and further organised following the piloted data extraction guidelines (Table 2). Extracted data was established *a priori*, involving (1) author(s), (2) study design, (3) Sample Size, (4) measurement tools, and (5) outcome of interest(s) for preclinical studies (Table 2). An additional category, diagnosis, was included for clinical studies (Table 3). Two reviewers (C.S. and Y.J.Z.) extracted data from relevant literature, followed up with thorough discussion to resolve potential conflicts. Alcohol consumption and outcome of interest(s) involved GLP-1 or GLP-1RA administration in the context of alcohol-related behaviours in preclinical and clinical literature.

**Table 2.** Characteristics of preclinical animal studies examining association between GLP-1 and alcohol-related behaviour

Author(s)	Study Type	Sample Size	Measurement Tools	Outcome(s) of Interest
Allingbjerg *et al*. (2023)	Animal Study	Male C57BL/6NTac mice (*n* = 31)	Blood Alcohol Levels Alcohol Self-Administration	Exendin-4 (Ex-4) micro-administration (i.e., vehicle, 3.2, 10, 32 ng/hemisphere) in the hippocampus and lateral septum (LS) reduced alcohol self-administration in mice and with large effect size, aligning with effects observed with systemic administration. Additionally, administration of exendin-4 into the nucleus accumbens reduced alcohol self-administration as reflective of previous studies. On the contrary, infusion of exendin-4 into the caudate-putamen (i.e., dorsal striatum administration of CPu group) did not show significant effect, likely due to the lack of GLP-1 receptor expression in this region. Overall, this study indicates that targeting GLP-1 receptors through its agonist results in a significant reduction in alcohol self-administration. Relative to vehicle group, Ex-4 administration reduced self-administration when micro-infused into the ventral hippocampus (p < .0001), the NAc, *F* (3, 21) = 4.46, *p* = 0.03, and the LS, *F* (3, 21) = 8.17, *p* = .001. Post hoc comparisons with the vehicle group revealed significant reductions at doses of 0.01 and 0.03 ng/hemisphere in both the NAc (*p* = 0.047 and *p* = 0.049) and the LS (*p* = 0.03 and *p* = 0.006). However, when administered to the CPu at these same doses, Ex-4 did not produce consistent or dose-dependent effects on alcohol self-administration compared to the vehicle group, as indicated by [*F* (3, 18) = 0.89, *p* = 0.44].
Aranas *et al*. (2023)	Animal Study	Rats (RCC/ Han Wistar) **Semaglutide on alcohol intake** male (*n* = 24), female (*n* = 24) **On relapse-like drinking** Male (*n* = 33), female (*n* = 31)	Alcohol-induced locomotor stimulation Dopamine release in NAc Conditioned place preference (CPP) test	Acute and repeated Semaglutide treatment does decrease alcohol intake in both male and female rats. More alcohol intake reduction was observed in males, and it suggests a relationship between alcohol intake and body weight. An overall interaction was observed (*F* (8,176) = 7.81, *P* < 0.0001), time (*F* (8,176) = 24.43, *P* < 0.0001) and tend toward treatment effect (*F* (1,22) = 3.86, *P* = 0.0623) on alcohol consumption. In males, semaglutide treatment did not alter water intake or total fluid intake, however for females, it did increase both water and total fluid intake. The 0.052 mg/kg dosage reduced alcohol consumption more than the 0.026 mg/kg. In each drinking session, semaglutide was found to significantly reduce alcohol intake at *P* < 0.0001. Semaglutide treatment also prevents alcohol consumption after withdrawals (*t* (14) = 2.67, *P* = 0.0187, paired *t*-test, *n* = 15). The treatment blocked relapse-like drinking, a significant reduction was observed at the 24th and 48th hour. It also reduced alcohol-induced dopamine activation in the mesolimbic dopamine system in the mice. Study also found semaglutide in the Nucleus accumbens shell in alcohol-drinking rats; it is able to enhance dopamine metabolism in alcohol-drinking rats and enhance dopamine driven behaviours in non alcohol-drinking mice.
Chuong *et al*. (2023)	Animal Study	**Mice** Male (*n* = 40) Female (*n* = 37) **Rats** Male (*n* = 21) Female (*n* = 21)	**Mice** Drinking-in-the-dark test (DID) Rotarod test Circular corridor test **Rats** Operant self-administration test Open-field test	Semaglutide significantly reduced sweet alcohol intake in mice (male *n* = 8, females *n* = 7) across all doses tested (*P* < 0.0001). It was also found to reduce unsweetened alcohol intake (*P* < 0.0001) with reductions at doses of 0.003 mg/kg (*P* = 0.05), 0.01 mg/kg (P = 0.0007), 0.03 mg/kg (*P* < 0.0001), and 0.1 mg/kg (*P* < 0.0001). Although females drank more alcohol, there was no dose × sex interaction was observed. Semaglutide also significantly reduced intake of sweet caloric fluids (*F* (5,65) = 5.53, *P* = 0.0003), water consumption (F(5,35) = 18.64, *P* < 0.0001), and non-caloric saccharin solutions. Semaglutide also significantly reduced binge-like alcohol drinking in both rats and mice. The main effect was observed at dose *F* (5,35) = 18.64, *P* < 0.0001 in non-independent rats, and reductions were observed across all doses. In alcohol-dependent rats, a main effect of dose (*F* (3,60) = 11.24, *P* < 0.0001) was observed, with a dosage of 0.1 mg/kg (P = 0.0007). No sex difference effects were observed.
Colvin *et al*. (2020)	Animal Study	Rats (*n* = 64 total)	Two-bottle choice paradigm Operant conditioning Paradigm	In a two hour period, Ex-4 significantly reduced alcohol consumption at doses 0.05 µg and 0.5 µg in the VTA (*F* (3,21) = 50.7, *p*<0.0001). In the Nucleus Accumbens Core (NAcC) and shell (NAcS), Ex-4 significantly decreased alcohol consumption, NAcC, *F* (3,21) = 37.7, *p* < 0.001; NAcS *F* (3,21) = 34.6, *p* < 0.001. In the Basolateral Amygdala (BLA), Arcuate Nucleus (ArcN), and Paraventricular Nucleus (PVN), Ex-4 had no significant impact on alcohol intake (p > 0.05). In the Dorsomedial Hippocampus (DMHipp), only the highest dosage, 0.5 µg, evoked a significant reduction in alcohol consumption (F (3,21) = 22.1, *p* < 0.01). **Operant conditioning paradigms:** In the Ventral Tegmental Area (VTA), ANOVA indicated that Ex-4 significantly reduced operant responding for palatable food rewards (F (3,21) = 62.8, *p* < 0.0001). Ex-4 also reduced operant in NAcS, NAcC, DMHipp, ArcN, PVN, and LH: *F* (3,21) = 41.6, *p* < 0.001; NAcS: *F* (3,21) = 52.7, *p* < 0.0001, *F* (3,21) = 38.9, *p* < 0.001, ArcN: *F* (3,21) = 40.2, *p* < 0.001; PVN: *F* (3,21) = 36.8, *p* < 0.001; LH: *F* (3,21) = 50.7, *p* < 0.0001. However, Ex-4 treatment did not alter operant responding for palatable food rewards *F* (3,21) = 3.4, *p* > 0.05.
Díaz-Megido and Thomsen (2023)	Animal Study	Male/Female C57BL/6J mice Male (*n* = 34) Female (*n* = 39)	Alcohol Self-administration (Operant-conditioning) Rotarod test	Alcohol reinforcers earned and estimated intake at baseline varied by day [F(4,236) = 6.36, *p* < 0.0001; *F* (4,236) = 5.25, *p* = 0.0005], with no sex effect (*p* = 0.6 and *p* = 0.3). Alcohol self-administration appeared more stable in males, but differences were not significant on individual days. Experiment 2 found no significant effects of time, sex, or their interaction in baseline measures (*p* = 0.2 − 0.5). **Experiment 1 cue-induced reinstatement of alcohol seeking:** Following systemic Ex-4 (saline, 1.8, and 3.2 μg/kg), female mice displayed condition-dependent nose poking (baseline, extinction, or reinstatement) ([F(2,54) = 64.6, *p* < 0.0001]) but there was no impact from Ex-4 treatment or interaction effects (*p* > 0.4). Post hoc tests revealed increased responses in the reinstatement test compared to extinction across all dose groups. Inactive nose-poke responses decreased from baseline to extinction, as shown by a significant condition effect ([F(2,52) = 11.5, *p* < 0.0001]), independent of treatment or interaction (*p* > 0.5). Further post hoc analysis confirmed lower inactive responses in extinction and reinstatement vs. baseline (*p* < 0.0001), with no difference between reinstatement and extinction (*p* > 0.8). Male mice displayed a relation between nose poking behaviour and condition [F(2, 42) = 70.8, *p* < 0.0001], and showed a significant treatment by condition interaction [F(4, 42) = 4.15, *p* = 0.006]. Overall, female mice displayed reinstatement of alcohol seeking with or without Ex-4 treatment, whereas Ex-4 inhibited reinstatement of alcohol seeking in male mice. During the cue-induced reinstatement test, analysis showed significant effects of Ex-4 dose [F(2,43) = 5.20, *p* = 0.01] and a sex-by-dose interaction [F(2,43) = 4.87, *p* = 0.01], though the main effect of sex was not quite significant [F(1,43) = 3.84, *p* = 0.056]. The interaction was driven by male mice, where Ex-4 significantly affected response [F(2,16) = 4.57, *p* = 0.03], while females showed no dose effect (*p* > 0.5). In males, both Ex-4 doses reduced reinstatement response compared to saline (*p* < 0.05). Responses in the inactive nose-poke hole were unaffected by sex, dose, or their interaction (*p* > 0.4). **Experiment 2 oral alcohol self-administration**: A follow-up analysis by sex revealed that Ex-4 dose significantly reduced alcohol self-administration in males [F(2,18) = 5.80, *p* = 0.01], with both doses significantly lower than saline (*p* = 0.01, *p* = 0.006). In females, the dose effect was not significant (*p* = 0.25). Responses in the inactive hole showed no significant effect of sex, dose, or their interaction (*p* > 0.36). To examine possible sex differences in pharmacokinetics, self-administration data were analysed separately for the first and second hours. Ex-4 showed significant effects in the first hour [F(2,31) = 9.75, *p* = 0.0005], with no impact of sex (*p* = 0.43) or interaction (*p* = 0.21). In males, Ex-4 significantly reduced self-administration [F(2,18) = 8.13, *p* = 0.003], with both doses lower than saline (*p* = 0.02, *p* = 0.0009). In females, the effect was not significant (*p* = 0.08). No significant effects were observed in the second hour (*p* = 0.26 − 0.70). Baseline levels before each test day were similar across doses, with no dose-by-sex interaction (*p* > 0.7), though females showed a trend toward fewer reinforcers (*p* = 0.08). The baseline day immediately after testing indicated a possible carryover effect of Ex-4, with a trend for dose effect [F (2,32) = 2.50, *p* = 0.098], independent of sex (main effect and interaction *p* > 0.8).
Dixon *et al*. (2020)	Animal Study	Male Long-Evans rats (*n* = 22 total)	Alcohol Self-administration **(Experiment 1)** Reacquisition **(Experiment 2)** PR/Motivation **(Experiment 3)**	**Experiment 1:** Rats exhibited a significantly higher number of reinforced active nose pokes compared to non-reinforced time-out pokes, regardless of whether they received intra-VTA treatment with vehicle (t (21) = 8.469, *p* < 0.01), 0.01 µg Ex-4 (t (21) = 7.037, *p* < 0.01), or 0.05 µg Ex4 (t (21) = 8.895, *p* < 0.01). This finding suggests that the active nose pokes were specifically associated with alcohol consumption rather than an overall increase in locomotor activity or a heightened incentive related to the nose poke apparatus. Analysis revealed a main effect of Ex- 4 treatment on non-reinforced nose pokes during time-out [F (2, 42) = 4.206, *p* < 0.05] and a reduction in alcohol infusion [F (2, 42) = 4.424, *p* < 0.05], with no significant dose-dependent differences. Alcohol consumption in g/kg also showed a treatment effect [F (2, 40) = 4.181, *p* < 0.05]. **Experiment 2:** Ex-4 administration yielded main effect on nose pokes [F (1, 21) = 58.185,p < 0.01], but no effect on treatment, [F (1, 21) = 0.075, *p* > 0.05], or nose pokes 9 treatment interaction, [F (1,21) = 0.257, *p* > 0.05], during reacquisition. Additionally, there were no significant effects observed on alcohol deliveries [t (21) = 0.773, *p* > 0.05] during acquisition. **Experiment 3:** VTA Ex-4 administration demonstrated no significant effect on motivation to seek out alcohol. Paired t tests showed no significant difference in the amount of alcohol deliveries [t (21) = 0.666, *p* > 0.05], relative to vehicle group. Prior findings indicate that Ex-4 more effectively reduces alcohol intake in high-alcohol-drinking (HAD) compared to low-alcohol-drinking (LAD) male Wistar rats. To assess this, a median split of baseline vehicle responses was analysed using a mixed model approach. Significant treatment × group effects revealed that Ex-4 selectively reduced alcohol self-administration in HAD rats, leading to decreases in active nose pokes, alcohol deliveries, and g/kg consumption at both 0.01 µg and 0.05 µg doses. LAD rats showed no notable response to Ex4, emphasising its specific efficacy in HAD rats.
Egecioglu *et al*. (2013)	Animal Study	Male NMRI mice Male Rcc Han Wistar rats	Alcohol Self-administration Conditioned Place Preference (CPP) Two-bottle-choice drinking paradigm	A higher dose of Ex-4 (4.8 μg/kg) significantly reduced (*P* < 0.01) alcohol-induced locomotor stimulation and further attenuated alcohol-induced dopamine release in the nucleus accumbens (*P* < 0.01 to *P* < 0.001). In CPP experiments, Ex-4 (2.4 μg/kg) significantly reduced alcohol-induced CPP (*P* < 0.01), but Ex-4 alone did not alter CPP in vehicle-conditioned mice (*P* > 0.05). Following repeated CPP, EX-4 administration throughout conditioning inhibited the effect of alcohol on CPP (*P* < 0.05) and co-treatment with Ex-4 in non-alcoholic conditioned mice did not induce CPP (*P* > 0.05). In high alcohol consuming rats, Ex-4 (1.2 μg/kg) significantly reduced alcohol intake at varying time points: at one hour [F (2,19) = 18.69, *P* < 0.001]; at four hours [F (2,19) = 12.95, *P* < 0.001]; and at 24 hours [F (2,19) = 8.236, *P* < 0.01]. However, 0.03 μg/kg dose showed no significant effect. In operant self-administration test, Ex-4 (1.2 μg/kg) resulted in reduction in active lever presses (*P* < 0.01) in rats following nine months of alcohol exposure.
Fink-Jensen *et al*. (2024)	Animal Study	Male Vervet Monkeys **Dose-finding** (*n* = 3) **Vehicle control** (*n* = 20)	Alcohol Consumption	During baseline, alcohol intake was similar between the vehicle and semaglutide (**dosages:** 0.01 mg/kg semaglutide for week 1, 0.03 mg/kg semaglutide for week 2, and 0.05 mg/kg for week 3) [F (1,18) = 2.058, *P* = 0.1686]. Following up-titration, semaglutide-treated animals consumed significantly less alcohol than vehicle-treated animals [F (1,18) = 9.939, *P* = 0.0055], with effects varying by week [F (2.709,48.76) = 6.441, *P* = 0.0013] and a treatment-week interaction [F (3,54) = 5.207, *P* = 0.0031]. Alcohol intake was significantly reduced in weeks one (*P* = 0.0428) and two (*P* = 0.0022), but not at week three (*P* = 0.0638). No difference in alcohol intake was observed during washout (*P* = 0.9703).
Liu *et al*. (2024)	Animal Study	**Experiment 1** Mice (*n* = 48) **Experiment 2:** Mice (*n* = 72)	Two-bottle choice alcohol drinking paradigm Open-Field test Elevated Plus Maze Morris Water Maze	After 6 weeks of exposure, liraglutide treatment significantly decreased alcohol consumption and preference in alcohol-treated mice compared to the alcohol-only group. After a two-week withdrawal from alcohol, mice treated with liraglutide consumed and preferred less alcohol, compared to the alcohol group during the re-drinking phase. No significant difference in the total fluid intake was observed between the two groups.
Marty *et al*. (2020)	Animal Study	Rats (*n* = 12)	Intermittent Access 2-Bottle Choice Drinking Paradigm, Intake and body weight measurements	GPR119, a GPCR, promotes GLP-1 secretion. GPR119 activation through the agonists AR231453 and APD668 did not significantly alter ethanol intake. However, Activation of GLP-1R does significantly reduce ethanol intake; both liraglutide and semaglutide did decrease ethanol intake and preference, though the effects were transient (liraglutide: treatment × time-point interaction: [F (2,22) = 47.5, *p* < 0.001]; semaglutide: treatment × time-point interaction: [F (2,22) = 65.75, *p* < 0.001]. However, the effects of both liraglutide and semaglutide did not last longer than 2 days post injection. It was also suggested that instead of having a direct effect on alcohol, liraglutide induced increased water intake, potentially responsible for reducing EtOH preference. Contrastingly, as semaglutide was found to significantly decrease EtOH preference on injection day, it appears to be more selective for EtOH as it did not affect water intake.
Shirazi *et al*. (2013)	Animal Study	**Mice** NMRI in CPP test (*n* = 48) **Rats:** GLP-1 on ethanol intake (*n* = 7) EX4 on ethanol intake (*n* = 9) Peripheral injection of GLP-1 (*n* = 24) Blockade of GLP-1 receptors (EX4) (*n* = 12-13)	Conditioned place preference (CPP) test Intermittent-access drinking model Microinjection studies	Study finds that peripheral injection of GLP-1 significantly reduces alcohol ingestion (nearly 30% less). Similarly, peripheral EX4 injection also showed a significant reduction in alcohol intake without altering general fluid intake. The efficacy of GLP-1 was more pronounced in rats with high alcohol intakes. During the CPP test, mice injected with alcohol displayed a significant preference for alcohol-associated compartments, whereas mice treated with GLP-1 did not. This indicates the blockage GLP-1 has on viewing alcohol as a reward.
Sørensen *et al.* (2016)	Animal Study	C57BL/6J mice (*n* = 14 total, 7 per cohort)	Operant Conditioning Chamber EtOH self-administration Ex-4 testing Food control test	Pre-treatment of 3.2 μg/kg Ex-4 significantly reduced alcohol consumption compared to baseline levels (*p* < 0.01). Due to the limitations in the experiment, EtOH intake was cut off at 30 reinforcers under baseline conditions in some mice, suggesting that the actual effect Ex-4 has on alcohol consumption might be greater than reported. After Ex-4 treatment, a representative mouse’s behaviour changed significantly, resembling a reduction of alcohol- seeking actions. The rate of response to alcohol reinforcement decreased to 16% of baseline.
Suchankova *et al*. (2015)	Animal Study	C57BL/6 mice (*n* = 72 to 80)	Two-bottle choice test Alcohol vapour inhalation chambers Forced-abstinence periods	AC3174 is an exenatide analogue, a modified version of Ex-4, which is a GLP-1R agonist. Prior to AC3174 treatments, EtOH (alcohol-dependent) mice consumed significantly more alcohol than control mice [F (1,67) = 13.61, *P* < 0.0001]. In test cycle 7, all AC3174 doses significantly reduced alcohol drinking compared with the EtOH mice injected with the vehicle. However, AC3174 treatment did not affect alcohol consumption in non-dependent CTL mice. After stopping AC3174 treatment, EtOH mice that received doses of 0.10 or 0.30 μg/kg, continued to consume significantly less alcohol [F (1,64) = 38.61, *P* < 0.00001] compared to vehicle mice. Before AC3174 treatment, ANOVA failed to show a main effect of treatment or group × treatment interaction. After AC3174 treatment, ANOVA shows *F* (1,65) = 31.44, *P* < 0.00001 for the main effect of the group, and [F (3,65) = 4.01, *P* < 0.025] for the group × treatment interaction, indicating alcohol dependence. During the washout period, ANOVA showed a significant main effect of the group [F (1,64) = 38.61, *P* < 0.00001], and [F (3,64) = 4.14, *P* < 0.01] for the group × treatment interaction. During the second washout period, EtOh mice still consumed more alcohol than CTL mice (F (1,64) = 16.30, *P* < 0.001 for the main effect of the group), however no significant treatment effect was observed.
Thomsen *et al*. (2017)	Animal Study	C57BL/6NTac mice (*n* = 24 total)	Alcohol Administration	Following the alcohol deprivation period, alcohol consumption and preference was significantly increased compared to baseline in the saline group but not in the Ex-4 group. ANOVA showed a significant treatment × time interaction for alcohol intake [F (8,131) = 5.02, *p* < 0.001] and effects of time [F (8,129) = 5.8, *p* < 0.001] and treatment [F (1,13) = 6.6, *p* < 0.05] on alcohol preference. Post-hoc analysis revealed increased alcohol intake and preference in the saline group post-deprivation (*p* ≤ 0.01, days 11–18 vs. baseline), with no change in the Ex-4 group. Ex-4 significantly reduced intake and preference compared to saline (*p* < 0.05, days 11–18). Upon Ex-4 discontinuation, both measures normalised to saline levels within a day, with no group differences during washout (days 19–25). During the post-deprivation period, Ex-4 significantly reduced the number of alcohol drinking bouts [F (1,13) = 11.6, *p* < 0.01] and delayed the first alcohol drink compared to saline (χ^2^ = 5.13, *p* < 0.05). Only 1 of 9 Ex-4 mice chose alcohol first, versus 5 of 6 in the saline group. Ex-4 mice also showed a trend toward larger water bout sizes [F (1,13) = 9.33, *p* < 0.01], with no differences in drinking bout number, duration, or latency to first water drink. Drinking patterns remained consistent across the 8-day period.
Thomsen *et al*. (2018)	Animal Study	Monkey (*n* = 32 total)	Drinking behaviour recording/ Alcohol consumption Gas chromatography Blood Sampling Behavioural Monitoring	Alcohol consumption significantly decreased in exenatide-treated monkeys during week one of alcohol reintroduction [F (1,21) = 6.80, *p* = 0.02], however, this reduction did not sustain into the second week. No differences in water intake were observed between the exenatide- and control group [F (4,84) = 5.87, *p* = 0.0003]. This suggests that exenatide only temporarily reduced alcohol intake, but no long-term impact was observed. Liraglutide was found to significantly lower alcohol intake compared to the vehicle group [F (1,22) = 17.3, *p* = 0.0004]. Alcohol intake was related to day [F (11,242) = 5.08, *p* < 0.0001], and a treatment by day interaction [F (11,242) = 2.44, *p* = 0.007], suggesting that liraglutide has a prolonged impact on lowering alcohol intake. In the liraglutide group, blood alcohol concentrations were significantly lower compared to the vehicle group (*p* < 0.05). Once liraglutide was withdrawn, a rebound effect was briefly observed before intake resumed to vehicle levels and the main effect of the treatment on alcohol consumption disappeared.
Vallof *et al*. (2015)	Animal Study	Male NMRI mice	Conditioned Place Preference (CPP) Dopamine Expression 20% alcohol two-bottle-choice drinking paradigm	Following systemic administration of liraglutide (0.1 mg/kg, subcutaneous), there was an attenuation of CPP and alcohol-induced dopamine release. In a drug-free test session after conditioning, alcohol-induced CPP (1.75 kg/kg) was significantly reduced by daily liraglutide (0.1 mg/kg) administration alongside alcohol on conditioning days compared to vehicle treatment (*P* = 0.0065, *n* = 7 per group). A control experiment confirmed no difference in CPP between liraglutide–vehicle and vehicle–vehicle treatments (*P* = 0.9788, *n* = 8 per group). However, a single post-conditioning dose of liraglutide (0.1 mg/kg) did not affect alcohol-induced CPP (*P* = 0.7903). Acute liraglutide (0.1 mg/kg, sc) in outbred rats (*n* = 15), after 12 weeks of intermittent alcohol consumption, yielded no significant difference in baseline alcohol intake between groups (vehicle: 4.0 ± 0.8 g/kg, *n* = 8; liraglutide: 3.4 ± 0.7 g/kg, *n* = 7; *P* = 0.5889). Liraglutide significantly reduced alcohol intake (*P* < 0.01), alcohol preference (*P* < 0.05), and food intake (*P* < 0.001) compared to the vehicle group. Additionally, the rats were categorised as high- or low-alcohol consumers based on baseline intake. Among high-alcohol-consuming rats, baseline alcohol intake (g/kg/24 hours) did not differ between those in the later receiving vehicle (3.9 ± 0.2, *n* = 9) or liraglutide (4.0 ± 0.2, *n* = 10; *P* = 0.7635). Liraglutide (0.05 mg/kg) significantly reduced alcohol intake (*P* = 0.0056) compared to vehicle. No differences were observed between treatments in water intake (*P* = 0.5891), total fluid intake (*P* = 0.0878), or alcohol preference (*P* = 0.1936). Furthermore, baseline alcohol intake (g/kg/24 hours) did not differ between low-alcohol-consuming rats designated for vehicle (*n* = 5) or liraglutide treatment (*n* = 4; *P* = 0.3235). In these low-alcohol-consuming rats, liraglutide (0.05 mg/kg) had no significant effect on alcohol intake (*P* = 0.1100) and alcohol preference (*P* = 0.5405) compared to vehicle treatment. In the next part of the study, the effect of acute liraglutide on relapse-like drinking was tested using an alcohol deprivation model in a separate group of rats (*n* = 11). Over 24 hours, treatment had a significant main effect [F (1,9) = 20.44, *P* = 0.0014] and a significant treatment × time interaction [F (1,9) = 11.39, *P* = 0.0082], though time alone was not significant [F (1,9) = 1.247, *P* = 0.2930]. Post hoc analysis showed a marked alcohol deprivation effect (increased intake vs. baseline) in vehicle-treated rats (*P* < 0.05), absent in liraglutide-treated rats (*P* > 0.05). Alcohol intake was significantly higher in vehicle-treated rats compared to liraglutide-treated rats (*P* < 0.001). Liraglutide treatment notably reduced alcohol intake (vehicle: 2.6 ± 0.4 g/kg; liraglutide: 0.6 ± 0.1 g/kg; *P* < 0.01) and preference (vehicle: 41 ± 7%; liraglutide: 12 ± 3%; *P* < 0.01). With 8 days of liraglutide treatment (0.1 mg/kg), including three alcohol-access days, significant treatment effects were seen for alcohol intake [F (1,13) = 5.838, *P* = 0.031] and time [F (2,26) = 4.642, *P* = 0.019], with liraglutide reducing intake in test sessions 1 and 2 (*P* < 0.05) but not 3. Alcohol preference was also lower with liraglutide [F (1,13) = 11.40, *P* = 0.0050] in sessions 1 and 2 (*P* < 0.01). Lastly, during the 5-day treatment phase, there were significant effects of treatment ([F (2,24) = 5.47, *P* = 0.0110]), time [F (4,96) = 3.15, *P* = 0.0176], and a treatment × time interaction [F (8,96) = 3.24, *P* = 0.0026] on alcohol-related lever responses. Similarly, self-administered alcohol was affected by treatment [F (2,24) = 3.46, *P* = 0.0479], time [F (4,96) = 3.92, *P* = 0.0054], and the interaction [F (8,96) = 4.15, *P* = 0.0003]. Post hoc analysis showed liraglutide (0.1 or 0.05 mg/kg) had no effect on day 1 compared to vehicle. By day 2, both doses reduced lever responses and alcohol intake by 40–50%. For days 3–5, responses in the 0.05 mg/kg group returned to control levels, while the 0.1 mg/kg group maintained reduced responses. In the 4-day post treatment, treatment had an overall effect on lever responses [F (2,24) = 6.52, *P* = 0.0055] and self-administered alcohol [F (2,24) = 4.56, *P* = 0.0209], with no significant time or interaction effects. Following treatment discontinuation, the 0.1 mg/kg liraglutide group maintained reduced responses for 2–3 days before returning to control levels.
Vallof *et al*. (2019a)	Animal Study	Male NMRI mice (high alcohol-consuming versus low-alcohol consuming)	Conditioned Place Preference (CPP) Intermittent access 20% alcohol two-bottle-choice drinking	**Ex-4 infusion into the (0.05 μg per side) NAc shell** Administration (*n* = 7) into the NAc shell resulted in attenuation of alcohol reward memory under the CPP paradigm [t (13) = 2.15, P = 0.0257]. However, there were no differences in CPP responses between control (*n* = 7) and Ex-4 (*n* = 6) mice following infusion in NAc shell [t (11) = 0.47, *P* = 0.3224]. High alcohol consuming rats showed no difference [t (13) = 0.29, *P* = 0.7787] following 12-weeks of baseline alcohol consumption (g/kg/24 hrs) in Ex-4 and vehicle rats. However, Ex-4 administration reduced alcohol intake [t (13) = 2.10, *P* = 0.0319] in these rats, but no difference in alcohol preference [t (13) = 1.17, *P* = 0.2615]. Low alcohol consuming rats showed no difference [t (5) = 0.76, *P* = 0.4836] following 12-weeks of baseline alcohol consumption (g/kg/24 hrs) in Ex-4 and vehicle rats. Ex-4 [t (5) = 0.20, *P* = 0.8518] did not show altered alcohol intake or alcohol preference [t (5) = 0.28, *P* = 0.7910] compared to vehicle group. **Ex-4 infusion into the (0.0025 μg per side) aVTA/pVTA and LDTg** **aVTA:** Alcohol reward memory in CPP were not different between Ex-4 (*n* = 8) and vehicle (*n* = 7) [t (13) = 0.85, *P* = 0.4111]. Additionally, Ex-4 administration showed no effect on alcohol intake [t (7) = 0.36, *P* = 0.7324] and preference [t (7) = 0.13, *P* = 0.9023] in high alcohol consuming rats (the only group present). **pVTA:** Likewise, CPP response did not differ between Ex-4 (*n* = 8) and vehicle (*n* = 7) [t (12) = 0.61, *P* = 0.5503]. Additionally, Ex-4 administration showed no effect on alcohol intake [t (7) = 0.10, *P* = 0.9219] and preference [t (7) = 0.05, *P* = 0.9644] in high alcohol consuming rats (the only group present). **LDTg:** Alcohol reward memory in CPP was not blocked by Ex-4 infusion (*n* = 23) [t (44) = 0. 54, *P* = 0.5914]. There were no distinctions in CPP response between vehicle (*n* = 7) and Ex-4 (*n* = 8) treatment following vehicle conditioning [t (13) = 0.81, P = 0.4329]. In this experiment, both low and high alcohol consuming rats were included in analysis. In low consuming rats, there was no distinction in 12 weeks baseline alcohol intake between rats that were treated with vehicles and Ex-4. Ex-4 treatment did not appear to affect alcohol consumption [t (13) = 0.53, P = 0.5270] nor alcohol preference [t (13) = 0.24, P = 0.8128]. Similarly, in high consuming rats, there was no difference in baseline alcohol intake between rats that were later treated with either vehicle or Ex-4. In this case, however, Ex-4 reduced alcohol intake [t (11) = 3.26, P = 0.0076] but did not affect alcohol preference [t (11) = 1.42, *P* = 0.1847].
Vallof *et al*. (2019b)	Animal Study	Vehicle (*n* = 17) Treatment 0.05μg (*n* = 15) 0.1μg (*n* = 11) 0.2μg (*n* = 10) Systemic administration of alcohol and intra-NTS injection of Ex-4 (*n* = 8)	Dopamine release measurements Locomotor activity registration Conditioned placement preference paradigm Intermittent access 20% alcohol two-bottle-choice drinking	Alcohol induces a locomotor response compared to the vehicle; the response is then inhibited by NTS-Ex-4 infusion at 0.05 μg per side. Ex-4 administration into NTS (nucleus of the solitary tract) shows that alcohol significantly increases locomotor activity compared to the vehicle (*P* < 0.001), the alcohol response was also significantly decreased in Ex-4 treated mice [*P* < 0.05 and *F* (3,49) = 2.85, *P* = 0.0469]. There was no significant difference in the locomotor activity between Ex-4 Alcohol-treated and vehicle mice (*P* > 0.05). A reduction in alcohol consumption in Ex-4 (0.05 μg per side) treated mice was observed at one, four, and 24 hrs, suggesting a consistent inhibitory effect of Ex-4. Alcohol preference significantly declined at the 24th hour, and water intake was not influenced at all. In the CPP test, Ex-4 treated mice showed a declined presence for the alcohol-associated compartment compared to controls (*P* < 0.05).
Vallof *et al*. (2020)	Animal Study	Rats About *n*≥5 per experiment, estimating about (*n* = 110 total)	Two-Bottle Choice Paradigm Body Weight Monitoring HPLC analysis for Biochemical Data	Both doses, 0.05 and 0.1mg/kg, decreased ethanol intake, however, baseline intake did not vary significantly [F (2,42) = 0.07, *P* = 0.9327]. Male rats were treated with dulaglutide treatment for 9 weeks, and ethanol intake and preference were observed to persist even after treatment was discontinued (*P* < 0.05), water and fluid intake did increase. In female rats, dulaglutide treatment did reduce ethanol intake in female rats, however this effect did not persist after the treatment ended (*P* < 0.05), water and total fluid intake was not affected. Dulaglutide was also found to reduce dopamine levels in both male and female rats, however it only increased noradrenaline in female rats. After a five-week treatment period, dulaglutide treatment was observed to reduce ethanol intake during the active treatment phase, and continued to persist even after discontinuation in male rats. In female rats, dulaglutide was found to reduce ethanol intake during the active treatment phase, but the effect did not persist after the treatment discontinuation.

**Table 3.** Characteristics of clinical studies examining association between glucagon-like peptide-1 and alcohol-related behaviours

Author(s)	Study Type	Sample Size	Diagnosis	Measurement Tools	Outcome(s) of Interest
Klausen *et al*. (2022)	Randomised Controlled Trial	Total (*n* = 127) 52.1 ± 10.8, and 60% men Exenatide (*n* = 62) Placebo (*n* = 65) 58 patients completed the trial	Alcohol Use disorders Identification Test (AUDIT)	Alcohol Consumption fMRI Alcohol Cue Reactivity	Exenatide did not significantly reduce the amount of heavy alcohol consuming days compared with the placebo group. However, there was significant attenuation of fMRI alcohol cue reactivity in the septal and ventral striatum, with lower dopamine transporter availability. Weekly exenatide administration did not reduce heavy drinking days in prespecified subgroups defined by baseline drinking days, DSM-5 severity, or geographic location. However, exploratory BMI subgroup analysis revealed that in obese patients (BMI>30 kg/m^2^, *n* = 30), exenatide significantly reduced heavy drinking days by 23.6% (95% CI [–44.4, –2.7], *P* = 0.034) and lowered total alcohol consumption by 1,205 g over 30 days (95% CI [–2,206,–204], *P* = 0.026) compared to placebo (Figs. 4 and 5). Conversely, in patients with BMI<25 kg/m^2^ (*n* = 52), exenatide increased heavy drinking days by 27.5% (95% CI [4.7, 50.2], *P* = 0.024) relative to placebo, though total alcohol intake did not differ between treatment groups. Additional post hoc analyses indicated no significant response differences by sex, baseline craving (Penn Alcohol Craving Scale), AUDIT score, baseline days abstinent, total baseline alcohol intake, or fMRI (*n* = 22) and SPECT (*n* = 16) subgroups. ROI analysis showed a significant treatment-by-time effect, with exenatide reducing cue reactivity in the ventral striatum, dorsal striatum, and putamen after 26 weeks compared to placebo. Specifically, cue-induced activity was significantly lower in the ventral striatum for the exenatide group (*P* = 0.025), while within-group reductions from baseline were also significant in the ventral and dorsal striatum for exenatide. Whole-brain analysis revealed initial cue reactivity differences in certain regions compared to healthy controls, but these differences resolved by week 26, with exenatide additionally reducing activation in the temporal lobe, hippocampus, and parahippocampus.
Quddos *et al*. (2023)	Retrospective Cohort Study	153 total participants	Alcohol Use disorders Identification Test (AUDIT)	Timeline follow back, Binomial model for binge drinking, Repeated measures analysis	In the remote clinical study, participants on Semaglutide and Tirzepatide showed significant reductions in alcohol consumption, binge drinking, and AUDIT scores. Additionally, reductions in the stimulative and sedative effects of alcohol were also reported; it reduced intoxication effects. Individuals with obesity on either Semaglutide or Tirzepatide had significantly fewer drinks compared to the non-medicated control group (*p* < 0.001). The impact was more pronounced in medication groups as their drinking frequency significantly decreased both on weekdays and weekends. The odds of binge-drinking alcohol according to the optimal binomial model states that Semaglutide vs. Control (*B* = −2.0517, *SE* = 0.6002, *p* < 0.001) and Tirzepatide vs. Control (*B* = −3.7920, *SE* = 0.6764, *p* < 0.001), suggesting that these medications do reduce the potential to binge-drink. A significant decrease in AUDIT scores were observed both within (pre- and post- starting medications) and between (medicated vs control) groups. The scores decreased after the participants started either medication. Drinks per episode of regular use also significantly decreased after the participants started taking their medication (*B* = 2.3443, *SE* = 0.2668, *p* < 0.001). The medications were also considered to reduce the alcohol’s intoxicating effects, stimulative (*B* = −9.057, *SE* = 1.623, *p* < 0.001) and sedative effects (*B* = −9.689, SE = 1.760, *p* < 0.001).

### Quality assessments

Quality assessment of pre-clinical studies was conducted using SYRCLE’s risk of bias analysis tool for animal studies, whereas quality assessment of clinical studies was conducted using Quality Assessment of Controlled Intervention Studies of the National Institute of Health (Hooijmans *et al*., 2014; Ma *et al*., 2020; National Institute of Health, 2013). Similarly, quality assessment of cohort studies was conducted using the Quality Assessment Tool for Observational Cohort and Cross-Sectional Studies (Ma *et al*., 2020; National Institute of Health, 2013). Independent reviewers (H.A., C.S., and Y.J.Z.) examined literature risk of bias and resolved all conflicts through further discussions. These tools were chosen for their established validity and reliability in assessing risk of bias in animal, clinical, and observational studies. The inclusion and exclusion criteria, comprehensive overview of data, and methodological quality are organised as supplementary materials (Table S1, S2).

## Results

### Search results

**Figure 1.**
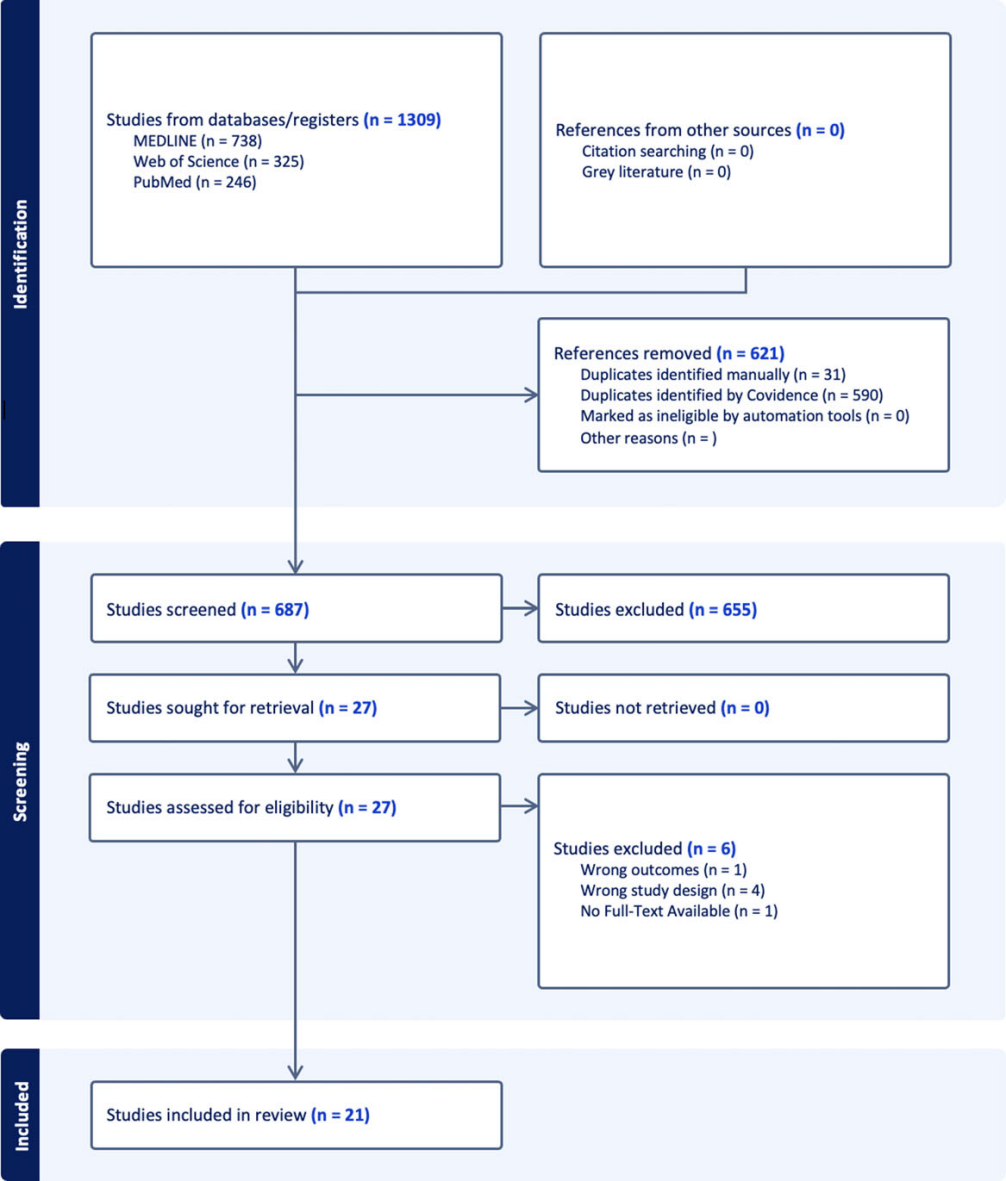
Preferred Reporting Items for Systematic Reviews and Meta-Analyses flow diagram of literature searches relevant to glucagon-like peptide-1 receptor agonists and alcohol consumption in preclinical and clinical studies (Systematic review management, 2023).

A systematic search of relevant literature yielded 1,309 studies. Among these, 31 duplicates were identified manually, and 590 were identified by Covidence (Fig. 1). 688 studies were screened based on their titles and abstracts according to the inclusion and exclusion criteria (Table 1). Following this, 28 full-text studies were assessed, with 21 studies deemed relevant and included for data extraction (Table 2). 6 studies were excluded, consisting of studies with incorrect study designs (*n* = 4), without available full text (*n* = 1), and with wrong outcomes (*n* = 1). Ultimately, 21 studies meeting the eligibility criteria were included in this systematic review (Table 2).

### Methodological quality

All controlled intervention studies were described as double-blinded, randomised trials, and had sufficient sample size to detect a clinically significant effect. Included observational cohort studies had sufficient sample size to detect a clinically significant effect, whilst employing consistent exposure measures across all study participants. Notwithstanding, Quddos *et al*. (2023) did not clearly specify the study population, and did not employ random assessment (Table S2). As such, we only examined the objective results investigating the association between GLP-1 RA administration and alcohol consumption behaviour for this study.

Most animal studies employed similar groups at baseline and/or adjusted for confounders and randomly housed animals during the experiment. Common limitations included the lack of blinding and random outcome assessment. Most studies did not exhibit a high degree of bias. Notwithstanding, studies rated ‘fair’ generally exhibited higher levels of categories marked as ‘Not Reported’ or ‘Not Applicable’, further omitting information regarding allocation concealment and insufficiently addressing incomplete outcome data (Table S1). Albeit the presence of limitations, the qualities of results were not affected.

### GLP-1 and GLP-1RA effect on alcohol-related behaviors

Cumulatively, we identified 21 studies investigating the role of GLP-1RA on alcohol consumption or administration in preclinical (*n* = 19) and clinical (*n* = 2) studies. The GLP-1RAs identified and assessed in this review are exenatide, semaglutide, and liraglutide.

### Preclinical evidence on the influence of GLP-1RA on alcohol-related behaviors

To understand the influence of GLP-1RAs (i.e., exenatide, semaglutide, and liraglutide) on alcohol consumption, 19 animal studies, consisting of rodents and monkey models, were identified. These studies used intracerebral injections to target varying brain regions, as well as peripheral infusion, to assess alcohol-associated behaviours.

### The role of exenatide on alcohol-related behaviors in preclinical studies

We identified 13 studies evaluating the impact of exenatide on alcohol consumption, demonstrating that exendin-4 (Ex-4) administration attenuates alcohol-related behaviours across various brain regions (Colvin *et al*., 2020; Allingbjerg *et al*., 2023; Díaz-Megido & Thomsen, 2023; Egecioglu *et al*., 2013; Dixon *et al*., 2020; Shirazi et al. 2013; Sørensen *et al.*, 2016; Suchankova *et al*., 2015; Thomsen *et al*., 2017; Thomsen *et al*., 2018; Vallof *et al*., 2019a; Vallof *et al.*, 2019b).

Allingbjerg *et al*. (2023) administered Ex-4 at varying doses (i.e., vehicle, 3.2, 10, 32 ng/hemisphere) in the NAc, ventral hippocampus (HPC), and lateral septum (LS), resulting in attenuated alcohol self-administration in C57BL/6J mice with a substantial effect size. Specifically, when micro-infused into the ventral HPC, Ex-4 significantly decreased self-administration compared to the vehicle group (*p* < .0001). Similar reductions were observed following administration to the NAc, *F* (3, 21) = 4.46, *p* = 0.03, and lateral septum (LS), *F* (3, 21) = 8.17, *p* = 0.001. Post hoc analyses further supported that doses of 0.01 and 0.03 ng/hemisphere in both the NAc and LS significantly reduced alcohol self-administration compared to vehicle (NAc: *p* = 0.047 and *p* = 0.049; LS: *p* = 0.03 and *p* = 0.006). A higher dose of Ex-4 (4.8 μg/kg) significantly reduced alcohol-induced locomotor stimulation and attenuated alcohol-induced dopamine release in the NAc (*P* < 0.01 to *P* < 0.001), highlighting its impact on both behavioural and neurochemical responses to alcohol (Egecioglu *et al*., 2013).

Additional findings by Colvin *et al*. (2020), support that Ex-4 administration into the ventral tegmental area (VTA) significantly reduced alcohol intake at 0.05 µg and 0.5 µg (*F* (3,21) = 50.7, *p* < 0.0001), aligning with reductions observed in the NAc core and shell (NAcC: *F* (3,21) = 37.7, *p* < 0.001; NAcS: *F* (3,21) = 34.6, *p* < 0.001). In the Dorsomedial Hippocampus (DMHipp), only the highest dose of 0.5 µg produced a significant reduction in alcohol consumption *F* (3,21) = 22.1, *p* < 0.01), indicating a dose-dependent effect specific to this region.

To further support these findings, Dixon *et al*. (2020) demonstrated that intra-VTA injections of Ex-4 (i.e., vehicle, 0.01 μg, 0.05 μg) yielded reduction in alcohol-self administration, which was most prominent in high-alcohol Long-Evans rats. It appears that Ex-4 selectively reduced alcohol-seeking behaviour in rats with a demonstrated specificity toward high-alcohol-drinking (HAD) phenotypes. In Experiment 1, Ex-4 treatment significantly attenuated non-reinforced nose pokes (*F* (2,42) = 4.206, *p* < 0.05), alcohol infusions (*F* (2,42) = 4.424, *p* < 0.05), and total alcohol consumption (*F* (2,40) = 4.181, *p* < 0.05), indicating that active nose pokes were directly associated with alcohol consumption rather than non-specific increases in activity. However, in Experiment 2, Ex-4 had no impact on reacquisition behaviours, as evidenced by unchanged alcohol deliveries (*t* (21) = 0.773, *p* > 0.05), and in Experiment 3, Ex-4 administration in the VTA showed no effect on motivation to seek alcohol (*t* (21) = 0.666, *p* > 0.05). This lack of effect on reacquisition and motivation suggests that Ex-4’s influence is not on general alcohol-seeking motivation but rather specific to conditions of established high alcohol intake.

Likewise, in high alcohol-consuming rats, 1.2 μg/kg Ex-4 effectively reduced alcohol intake across multiple time points ([1 hour: *F* (2,19) = 18.69, *P* < 0.001; 4 hours: *F* (2,19) = 12.95, *P* < 0.001; 24 hours: *F* (2,19) = 8.236, *P* < 0.01]), whereas a lower 0.03 μg/kg dose had no significant effect (Egecioglu *et al*., 2013). Additionally, in an operant self-administration test, 1.2 μg/kg Ex-4 significantly reduced active lever presses (*P* < 0.01) in rats with prolonged alcohol exposure, indicating its potential to suppress established alcohol-seeking behaviours.

In conditioned place preference (CPP) experiments, a 2.4 μg/kg dose of Ex-4 significantly reduced alcohol-induced CPP (*P* < 0.01) without affecting CPP in vehicle-conditioned mice (*P* > 0.05), demonstrating specificity for alcohol-related cues. When administered throughout conditioning, Ex-4 inhibited alcohol’s effect on CPP (*P* < 0.05), and co-treatment in non-alcohol-conditioned mice did not induce CPP (*P* > 0.05). An increased dose (i.e., 3.2 μg/kg Ex-4) further reduced alcohol consumption relative to baseline (*P* < 0.01) (Sørensen *et al.*, 2016). Furthermore, mice response to alcohol reinforcement was reduced to 16% of the baseline, demonstrating reduction in alcohol-seeking behaviours.

Further evidence in accordance with the aforementioned findings are provided by Vallof *et al*. (2019a), wherein researchers infused Ex-4 into the NAcS in low and high alcohol consuming mice. Ex-4 infusion into the NAcS (0.05 μg per hemisphere) resulted in an attenuation of alcohol reward memory under CPP testing (*t* (13) = 2.15, *P* = 0.0257)). However, there were no distinctions in CPP responses between control and Ex-4 mice (*t* (11) = 0.47, *P* = 0.3224)). Additionally, low alcohol consuming rats showed no significant alterations (*t* (5) = 0.76, *P* = 0.4836)) following 12-weeks of baseline alcohol consumption in Ex-4 and vehicle rats (Vallof *et al*., 2019a). Locomotor response to alcohol was further inhibited by nucleus of the solitary tract (NTS)-Ex-4 administration at 0.05 μg per hemisphere at one, two, and 24 hours (*P* < 0.001) (Vallof *et al.*, 2019b). Alcohol response was significantly decreased in Ex-4 treated mice (*P* < 0.05, *F* (3,49) = 2.85, *P* = 0.0469). Consistent with existing literature, Ex-4 treated mice showed a declined presence for the alcohol-associated compartment compared to controls in CPP testing (*P* < 0.05) (Vallof *et al*., 2019b).

Alongside Ex-4’s disparate impact in varying drinking phenotypes (i.e., high versus low consumption), studies further characterised potential sex differences and alcohol-related behaviours following Ex-4 administration (Díaz-Megido & Thomsen, 2023). For cue-induced reinstatement, systemic Ex-4 treatment (saline, 1.8, and 3.2 μg/kg) had no impact on reinstatement responses in female mice, who showed condition-dependent nose poking [*F* (2,54) = 64.6, *p* < 0.0001] with a lack of treatment effect (*p* > 0.4) (Díaz-Megido & Thomsen, 2023). Post hoc analysis confirmed that reinstatement responses exceeded extinction, and inactive responses decreased from baseline to extinction (both *p* < 0.0001), with no significant differences between reinstatement and extinction (*p* > 0.8). In male mice, however, Ex-4 significantly reduced reinstatement behaviour, showing a dose-dependent response ([*F* (2,16) = 4.57, *p* = 0.03]).

Analysis further revealed a dose effect ([*F* (2,43) = 5.20, *p* = 0.01]) and a sex-by-dose interaction ([F (2,43) = 4.87, *p* = 0.01]), driven by male responses. Inactive nose-poke responses were unaffected by sex or dose (p > 0.4). Additionally, Ex-4 significantly reduced oral alcohol self-administration in males ([F (2,18) = 5.80, *p* = 0.01]), with both doses decreasing intake relative to saline group (*p* = 0.01, *p* = 0.006). Notwithstanding, female mice had no significant dose effect (*p* = 0.25). Additional analysis for the first- and second-hour following treatment indicated Ex-4 reduced male self-administration in the first hour ([*F* (2,18) = 8.13, *p* = 0.003), with both doses yielding lower alcohol-related behaviours than saline (i.e., *p* = 0.02, *p* = 0.0009). Female mice displayed a non-significant trend (p = 0.08), with no significant effects observed in the second hour (*p* = 0.26 − 0.70) (Díaz-Megido & Thomsen, 2023).

Correspondingly, Thomsen *et al*. (2017) identified a significant increase in alcohol consumption and preference compared to baseline in saline (*p* ≤ 0.01, days 11–18 vs. baseline) but not Ex-4 C57BL/6NTac mice groups, post-alcohol deprivation. Ex-4 significantly reduced alcohol intake and preference compared to saline (*p* < 0.05, days 11–18). In the post-deprivation period, Ex-4 significantly reduced the number of alcohol drinking bouts ([*F* (1,13) = 11.6, *p* < 0.01]) and delayed the first alcohol drink compared to the saline group (χ^2^ = 5.13, *p* < 0.05). Only one of nine Ex-4 mice chose alcohol first, versus five of six in the saline group. With Ex-4 discontinuation, both measures normalised to saline levels within a day, with no group differences during washout (days 19 to 25). Thomsen *et al*. (2018) followed up their research in monkeys, wherein alcohol consumption was significantly attenuated following exenatide treatment in week one of alcohol reintroduction (*F* (1,21) = 6.80, *p* = 0.02), but this effect was not preserved in week two.

With peripheral injection of Ex-4, there was also a significant reduction in alcohol consumption, without alterations in general fluid intake (Shirazi *et al*., 2013). Similar effects were observed in a modified version of Ex-4 (i.e., AC3174), wherein AC3173 doses significantly reduced alcohol consumption relative to vehicle EtOH mice (Suchankova *et al*., 2015). Prior to treatment, EtOH (alcohol-dependent) mice consumed significantly more alcohol than control mice (*F* (1,67) = 13.61, *P* < 0.0001). Following, AC3174 administration reduced alcohol drinking, but did not significantly affect alcohol consumption in non-alcohol-dependent control mice.

Literature further demonstrated that selective administration in brain regions is crucial for Ex-4 effectiveness, as certain brain regions may not be an optimal targeting site. Notably, Ex-4 did not yield significant effects when injected in the caudate putamen (CPu), basolateral amygdala (BLA), arcuate nucleus (ArcN), paraventricular nucleus (PVN), anterior VTA (aVTA), posterior VTA (pVTA), and lateral dorsal tegmental nucleus (LDTg) (Colvin *et al*., 2020; Allingbjerg *et al*., 2023; Vallof *et al*., 2019a). Ex-4 administration into the CPu, representing dorsal striatal targeting, did not produce significant alterations in alcohol self-administration behaviour, likely reflecting the lower density or absence of GLP-1Rs in this region [*F* (3, 18) = 0.89, *p* = .44] (Allingbjerg *et al*., 2023). Additionally, Ex-4 administration did not significantly influence alcohol intake in the BLA, ArcN, or PVN (*p* > 0.05) (Colvin *et al*., 2020). Research also identified lack of significant effect on alcohol-related behaviours in the aVTA, pVTA, and LDTg, wherein Ex-4 infusion (0.0025 μg per hemisphere) had limited effects on CPP response, alcohol consumption, and alcohol preference (Vallof *et al*., 2019a). In the aVTA, Ex-4 did not alter alcohol reward memory in conditioned place preference (CPP) compared to vehicle (t(13) = 0.85, *P* = 0.4111) and had no effect on alcohol intake (t(7) = 0.36, *P* = 0.7324) or preference (t(7) = 0.13, *P* = 0.9023) in high alcohol-consuming rats. Similarly, in the pVTA, Ex-4 did not affect CPP (*t* (12) = 0.61, *P* = 0.5503), alcohol intake (*t* (7) = 0.10, *P* = 0.9219), or preference (*t* (7) = 0.05, *P* = 0.9644) in high alcohol-consuming rats. In the LDTg, Ex-4 infusion had no effect on CPP in either low or high alcohol-consuming rats (*t* (44) = 0.54, *P* = 0.5914), with no differences in CPP between vehicle and Ex-4 groups in vehicle-conditioned rats (*t* (13) = 0.81, *P* = 0.4329). In low alcohol-consuming rats, Ex-4 had no effect on baseline alcohol intake (*t* (13) = 0.53, *P* = 0.5270) or preference (*t* (13) = 0.24, *P* = 0.8128). However, in high alcohol-consuming rats, Ex-4 significantly reduced alcohol intake (*t* (11) = 3.26, *P* = 0.0076) without affecting alcohol preference (*t* (11) = 1.42, *P* = 0.1847). These findings suggest Ex-4’s selective efficacy in reducing intake specifically in high-consuming phenotypes, with minimal effects on alcohol reward memory or preference across regions (Vallof *et al*., 2019a).

### The role of semaglutide on alcohol-related behaviors in preclinical studies

We identified 4 studies that reported on the effects of semaglutide on alcohol consumption in preclinical studies (Aranas *et al*., 2023; Marty *et al*., 2020; Chuong *et al*., 2023; Fink-Jensen *et al*., 2024). Aranas et al. (2023) reported that acute and repeated semaglutide treatment significantly decreased alcohol intake in both male and female rats, with more pronounced reductions in males, suggesting a potential link between alcohol intake and body weight (*P* < 0.0001). Higher reductions in alcohol consumption were observed with higher dosages, indicating a dose-dependent association. Furthermore, semaglutide was also found to prevent post-withdrawal alcohol consumption (t (14) = 2.67, *P* = 0.0187, paired *t*-test, *n* = 15) and relapse drinking, with significant reductions reported at the 24th and 48th hour. Similarly, Chuong *et al*. (2023) observed that semaglutide significantly reduced preference for both sweetened and unsweetened alcohol (*P* < 0.0001) across all doses, but significant reductions were reported for doses 0.003mg/kg, 0.01mg/kg, 0.03mg/kg, and 0.1mg/kg. While female rodents were found to consume more alcohol, no significant dose-sex interaction was reported. Despite the general efficacy of semaglutide, Fink-Jensen *et al*. (2024) found that while alcohol consumption was significantly less in semaglutide-induced mice than vehicle controls (*P* = 0.0055), reductions only lasted through the second week (week 1: *P* = 0.0428, week 2: *P* = 0.0022, week 3: *P* = 0.0632). This finding suggests that the drug’s effect may be short-lived. Its transient effects were also supported by Marty *et al*. (2020)’s findings, as researchers found that while semaglutide did reduce ethanol consumption and preference, the effects did not last more than two days post-drug administration. Furthermore, semaglutide demonstrated the ability to selectively reduce alcohol consumption without affecting other fluid intake, and these reductions were unaffected by the administration of Ex9-39, a GLP-1R antagonist, suggesting that its effects are independent.

### The role of liraglutide on alcohol-related behavior in preclinical studies

With respect to liraglutide treatment, four studies exploring its effect on alcohol consumption were identified (Thomsen *et al*., 2018; Marty *et al*., 2020; Liu *et al*., 2024; Vallof *et al*., 2015). These studies explore the effects liraglutide has on consumption and preference, and the complexities of its underlying mechanisms. Findings by Liu *et al*. (2024) reported that reductions in alcohol consumption and preference were observed in alcohol treated mice treated with liraglutide for 6 weeks in comparison to the control group. Notably, these mice continued to exhibit these effects (relative to the control group) even after a two-week alcohol withdrawal period. Interestingly, total fluid intake was unaffected in both groups, suggesting that the reduction in alcohol preference was due to a selective effect on alcohol rather than general changes in fluid intake.

Correspondingly, Marty *et al*. (2020)’s findings demonstrated that while liraglutide did reduce ethanol intake and preference (liraglutide: treatment × time-point interaction), the effects were transient and did not last for more than 2 days post-injection. The researchers proposed that, forasmuch as liraglutide may have increased water intake, it accounts for the reduction in alcohol preference as it may not have directly affected alcohol consumption.

In Thomsen *et al*. (2018)’s study, liraglutide was observed to significantly reduce alcohol consumption compared to the vehicle group (*F* (1,22) = 17.3, *p* = 0.0004). This intake was related to day (*F* (11,242) = 5.08, *p* < 0.0001), and a treatment by day interaction (*F* (11,242) = 2.44, *p* = 0.007), indicating that the treatment had a sustained impact on alcohol consumption until the second day, which is in accordance with Marty *et al* (2020)’s findings. Following the second day, a distinguishable rebound effect was briefly observed before alcohol intake resumed to vehicle-levels and the liraglutide treatment effect disappeared.

Vallof *et al*. (2015) focused primarily on the effects of liraglutide on alcohol-associated conditioned place preference (CPP) and alcohol-induced dopamine release. Liraglutide treatment at a dosage of 0.1mg/kg significantly reduced alcohol-induced CPP and dopamine release (*P* = 0.0065). Liraglutide was also found to significantly reduce both alcohol intake (*P* < 0.01) and alcohol preference (*P* < 0.05), compared to the vehicle group. In high-alcohol consuming rats, liraglutide was found to significantly reduce alcohol consumption (*P* = 0.0056), without affecting water and total fluid intake, or alcohol preference. Conversely, no significant effect on alcohol intake (*P* = 0.1100) and alcohol preference (*P* = 0.5405) was observed in low-alcohol consuming rats, relative to the vehicle treatment. In a relapse-like drinking model, marked deprivation effects associated with liraglutide’s effects on alcohol intake and preference were detected in control group rats and absent in the treatment group after 24 hours. While the reductions in alcohol intake appeared transient over time, the effects were prolonged with longer treatment periods. Significant reductions were prominent in the earlier sessions but soon returned to baseline levels in later days, especially with the 0.05mg/kg dosage. The 0.1mg/kg group experienced prolonged effect responses for 2-3 days before returning to vehicle-like levels.

### Clinical evidence on the influence of glp-1ra on alcohol-related behaviors

We identified two clinical studies evaluating the effects of GLP-1RAs (e.g., semaglutide and on alcohol consumption in patients diagnosed with AUD (Klausen *et al*., 2022; Quddos *et al*., 2023). Results from the clinical literature indicate that there are mixed conclusions drawn on the role of GLP1-RAs on alcohol-related behaviours (e.g., alcohol consumption).

In a randomised control trial by Quddos *et al*. (2023) (*n* = 153), semaglutide administration was associated with significant attenuation of alcohol consumption in comparison to control and non-treatment groups (*p* < 0.001). Notably, binge-drinking behaviours were reduced following treatment, as supported by the binomial model [i.e., Semaglutide vs. Control (*B* = −2.0517, *SE* = 0.6002, *p* < 0.001)]. Additionally, drinks per episode of regular usage were significantly reduced after participants initiated medication consumption (*B* = 2.3443, *SE* = 0.2668, *p* < 0.001). Quddos *et al*. (2023) further identified significant reduction in AUDIT scores within (i.e., pre- and post- starting medications) and between (i.e., medicated vs control) groups following semaglutide administration.

In an intervention study, Klausen *et al*. (2022) reported that weekly exenatide administration did not significantly reduce heavy alcohol consumption days relative to the placebo group. Notwithstanding, an exploratory BMI subgroup analysis indicates that exenatide significantly decreased heavy drinking days by 23.6% (95% CI [−44.4, −2.7], *P* = 0.034) and reduced cumulative alcohol consumption by 1,205g within a month (95% CI [−2,206, −204], *P* = 0.026) in obese patients (BMI>30 kg/m^2^, *n* = 30) relative to placebo group. However, these trends were not observed in patients with a BMI<25 kg/m^2^ (*n* = 52). Instead, exenatide administration increased heavy drinking days by 27.5% (95% CI [4.7, 50.2], *P* = 0.024) relative to placebo, though fMRI Alcohol Cue Reactivity analysis revealed a significant reduction in cue reactivity in ventral striatum, dorsal striatum, and putamen after 26 weeks. Whole-brain analysis further revealed initial cue reactivity differentiation in certain regions compared to healthy controls, but these differences were resolved by week 26, with exenatide additionally reducing activation in the temporal lobe, hippocampus, and parahippocampus. Nonetheless, there is not adequate information on the effects of GLP-1RAs on alcohol consumption and associated behaviours.

## Discussion

Extensive literature provides evidence associating GLP-1RAs activity and modulation of alcohol-related behaviours, mediated by their interaction with key neurobiological pathways (Shirazi *et al*., 2013; Colvin *et al*., 2020; Vallof *et al*., 2019a; Vallof et al., 2019b). Particularly, GLP-1RAs modulate the mesolimbic dopamine system, a critical circuit involved in reward and reinforcement processes, by targeting GLP-1R expressed in regions such as the NAc and VTA (Allingbjerg *et al*., 2023; Aranas *et al*., 2023; Colvin *et al*., 2020; Dixon *et al*., 2020; Vallof *et al*., 2019a). Furthermore, GLP-1RAs attenuate alcohol-induced dopamine release in theseregions, subsequently reducing alcohol preference, consumption, and associated reinforcing effects (Aranas *et al*., 2023; Egecioglu *et al*., 2013; Liu *et al*., 2024; Marty *et al.*, 2020; Shirazi *et al*., 2013; Thomsen *et al*., 2017; Vallof *et al*., 2015; Vallof *et al*., 2019a; Vallof *et al*., 2019b). As such, these agonists may influence alcohol-related behaviours through their effects on brain regions, including the hypothalamus, amygdala, and HPC, which have known effects on stress, memory, and emotional responses relative to alcohol cues. These mechanisms collectively support the therapeutic potential of GLP-1RAs in altering alcohol and reward-related behaviours in pre-clinical models and clinical population.

Despite evidence supporting GLP-1RA mediated modulation of alcohol-related behaviours, notable considerations remain. Particularly, pre-clinical studies demonstrate that the efficacy of GLP-1RAs in reducing alcohol-related behaviours is dependent on the administration site. Brain regions such as the NAc and VTA consistently yield significant reductions in alcohol consumption and preference, reflecting their central role in the reward circuitry (Colvin *et al*., 2020; Dixon *et al*., 2020; Allingbjerg *et al*., 2023; Vallof *et al*., 2019a). For instance, exenatide administered directly into the NAc or VTA effectively attenuates alcohol self-administration and dopamine release, highlighting these regions as optimal targets for GLP-1RA interventions. Conversely, GLP-1RA administration in regions such as the CPu, BLA, ArcN, and PVN appears less effective or altogether ineffective (Colvin *et al*., 2020; Allingbjerg *et al*., 2023; Vallof *et al*., 2019a). This may reflect lower GLP-1R density or limited involvement of these regions in alcohol reward pathways (Allingbjerg *et al*., 2023). These findings emphasise the importance of targeted administration strategies to maximise the efficacy of GLP-1RAs in preclinical models and potentially in clinical settings.

Another consideration is the influence of GLP-1RAs on alcohol-related behaviours varying across sex and drinking phenotypes. Male rodents generally exhibit greater reductions in alcohol consumption and preference following GLP-1RA administration compared to females, suggesting a sex-dependent response likely influenced by hormonal or neurochemical differences (Díaz-Megido & Thomsen, 2023). However, a few examined studies found no significant interaction between dose and sex, they observed a sex- independent relationship. Furthermore, GLP-1RAs appear more effective in high-alcohol-consuming phenotypes, reducing alcohol-related behaviours to a greater extent than in low-consuming counterparts. This variability emphasises the need for additional examinations into the biological underpinnings of these differences to inform personalised treatment approaches.

In addition, clinical studies evaluating GLP-1RAs for AUD patients demonstrated mixed findings. While semaglutide has demonstrated promising reductions in alcohol consumption and binge-drinking behaviours, the effects of exenatide appear to vary based on patient characteristics, such as body mass index). For instance, obese patients exhibited significant reductions in heavy drinking days and alcohol consumption with exenatide treatment, whereas non-obese patients showed minimal or even adverse effects. These contrasting results suggest that GLP-1RA efficacy may depend on individual variability, necessitating further investigation to identify patient subgroups most likely to benefit from treatment.

A recurring limitation observed in assessed preclinical and clinical studies is the transient nature of GLP-1RA effects on alcohol-related behaviours. While significant reductions in alcohol consumption and preference are often observed initially, these effects diminish over time (Marty *et al*., 2020; Fink-Jensen *et al*., 2024; Vallof *et al*., 2015), potentially due to receptor desensitisation or compensatory neuroadaptations. Receptor desensitisation and neuroadaptations may limit the long-term efficacy of GLP-1RAs. As such, future research should further evaluate strategies to mitigate tolerance, such as dose escalation protocols, intermittent dosing schedules, or combination therapies targeting complementary pathways.

Other possible explanations may stem from the intricacies of the neuroendocrine system, where prolonged modifications in reward pathways are counterbalanced by compensatory hormonal fluctuations. Furthermore, the progressive reinstatement of alcohol intake may be facilitated by various factors that are not sufficiently regulated by the pharmacological activity of GLP-1RAs. This limitation highlights the need for longitudinal studies to evaluate the sustained efficacy of GLP-1RAs and explore strategies to mitigate tolerance, such as dose adjustments or combination therapies.

Lastly, another notable limitation of the current literature (Erbil *et al*., 2019) is the variability in the neuroprotective effects of various GLP-1 analogues. Evidence suggests that these effects are largely dose-dependent, where higher doses are associated with more significant neurodegenerative process reversals; such effects could have profound and similar implications in reward pathways and neural circuits involved in neurodegeneration and substance abuse. Additionally, treatment combinations have been suggested to yield greater efficacy, however most of the reviewed studies did not explore the potential synergistic effects combining different analogues, which is an aspect that could have vital implications in understanding their impact on alcohol-related behaviours and reward pathways. The omission of such combination therapies in these studies results in a gap in research, warranting further exploration on therapeutic avenues regarding GLP-1RAs and addiction interventions.

## Conclusion

Results from assessed preclinical and clinical studies underscore the potential of GLP-1RAs in attenuating alcohol-related behaviours, though the extent and consistency of these effects vary across experimental models and patient populations. Extant preclinical studies suggest that GLP-1RAs (i.e., exenatide, semaglutide, and liraglutide) attenuate alcohol consumption, preference, and alcohol-induced neurochemical responses in rodent models, with nuanced differences influenced by dosage, administration route, and target brain regions. Notably, the efficacy of these agents appears selective of high-alcohol-consuming phenotypes and specific brain regions such as NAc and VTA. However, discrepancies in findings, including the transient nature of effects and region-dependent variability, emphasise the need for further mechanistic studies into GLP-1RA’s influence on alcohol-related pathways. Additionally, clinical evidence, while limited, has yielded mixed conclusions regarding the influence of GLP-1RAs on alcohol-associated behaviours. While semaglutide has shown promise in reducing alcohol consumption and binge-drinking episodes, the effects of exenatide appear to be influenced by patient characteristics such as BMI, suggesting variability in treatment response. These findings emphasise the need for additional clinical studies to clarify the therapeutic efficacy of GLP-1RAs in addressing AUD. Recent analysis on GLP-1 suggests an association with suicide, but with no causal effects (McIntyre *et al*., 2023; McIntyre, 2024; McIntyre *et al*., 2024; McIntyre *et al*., 2025). Further research should concentrate on elucidating the mechanisms underlying these effects, identifying biomarkers of treatment response, and optimising intervention strategies to enhance clinical applicability.

Lastly, this review does not examine the role of GLP-1R antagonists, inhibition techniques, or genetic knockouts to explicitly confirm whether observed effects on alcohol-related behaviours are directly mediated through GLP-1R signalling. Without these studies, it remains uncertain whether GLP-1RAs exert their effects through GLP-1 receptor activation or if alternative pathways contribute to the behavioural outcomes. As such, this gap necessitates further incorporation of antagonist, inhibitory, or genetic techniques to confirm the role of GLP-1R activation through GLP-1RAs in alcohol-associated behaviours. Future studies should integrate antagonist administration or genetic knockout models to delineate the precise role of GLP-1R signalling, clarifying mechanistic specificity and excluding confounding influences. Addressing this gap is essential for refining therapeutic strategies, optimising intervention efficacy, and identifying potential off-target mechanisms influencing alcohol-related behaviours.

## Supporting information

Zheng et al. supplementary material Zheng et al. supplementary material

## Supplementary material

The supplementary material for this article can be found at https://doi.org/10.1017/neu.2025.6.
