# Supplementary material for: A systematic review on the role of glucagon-like peptide-1 receptor agonists on alcohol-related behaviors: potential therapeutic strategy for alcohol use disorder
Source: Acta Neuropsychiatr. 2025 Feb 19;37:e51. doi: 10.1017/neu.2025.6 (PMC13130248; doi:10.1017/neu.2025.6)
Supplement: Zheng et al. supplementary material [file S0924270825000067sup001.docx]

**SUPPLEMENTARY MATERIALS**

**Table S1.** Risk of bias/quality assessment of the included studies using the SYRCLE’s tool for assessing risk of bias in animal studies (Hooijmans et al., 2014).

| **Study** | **Item** | | | | | | | | | | **Quality Rating** |
| --- | --- | --- | --- | --- | --- | --- | --- | --- | --- | --- | --- |
|  | **1** | **2** | **3** | **4** | **5** | **6** | **7** | **8** | **9** | **10** |  |
| Allingbjerg et al. (2023) | **✓** | **✓** | **✓** | **✓** | **X** | **✓** | **X** | **✓** | **✓** | **✓** | Good |
| Aranas et al. (2023) | **✓** | **✓** | **✓** | **✓** | **X** | **NR** | **X** | **NR** | **✓** | **✓** | Good |
| Chuong et al. (2023) | **✓** | **✓** | **NR** | **✓** | **NA** | **NA** | **NA** | **NA** | **NA** | **✓** | Good |
| Colvin et al. (2020) | **✓** | **✓** | **✓** | **✓** | **NA** | **✓** | **X** | **✓** | **✓** | **✓** | Good |
| Diaz-Megido et al. (2023) | **✓** | **✓** | **✓** | **✓** | **NR** | **✓** | **NR** | **NA** | **✓** | **✓** | Good |
| Dixon et al. (2020) | **NR** | **✓** | **✓** | **✓** | **NR** | **NA** | **NR** | **NR** | **✓** | **✓** | Fair |
| Egecioglu et al. (2013) | **✓** | **✓** | **✓** | **X** | **NR** | **NR** | **NR** | **NR** | **✓** | **✓** | Fair |
| Fink-Jensen et al. (2024) | **✓** | **✓** | **NR** | **X** | **✓** | **NR** | **✓** | **NR** | **✓** | **✓** | Good |
| Liu et al. (2024) | **✓** | **✓** | **NR** | **NA** | **X** | **✓** | **✓** | **✓** | **✓** | **✓** | Good |
| Marty et al. (2020) | **NA** | **✓** | **NA** | **X** | **X** | **NA** | **X** | **✓** | **✓** | **✓** | Fair |
| Shirazi at al. (2013) | **✓** | **✓** | **NR** | **NA** | **X** | **NR** | **X** | **NR** | **✓** | **✓** | Fair |
| Sorensen et al. (2016) | **✓** | **✓** | **✓** | **✓** | **NR** | **NR** | **NR** | **✓** | **✓** | **✓** | Good |
| Suchankova et al. (2015) | **NA** | **✓** | **NR** | **✓** | **NR** | **NR** | **NR** | **NA** | **✓** | **✓** | Fair |
| Thomsen et al. (2017) | **NR** | **✓** | **✓** | **✓** | **X** | **NA** | **X** | **✓** | **✓** | **✓** | Good |
| Thomsen et al. (2018) | **✓** | **X** | **✓** | **✓** | **✓** | **NR** | **✓** | **✓** | **✓** | **✓** | Good |
| Vallof et al. (2015) | **✓** | **✓** | **✓** | **NR** | **X** | **X** | **NR** | **✓** | **✓** | **✓** | Good |
| Vallof et al. (2019a) | **✓** | **✓** | **✓** | **✓** | **NR** | **NR** | **NR** | **✓** | **✓** | **✓** | Good |
| Vallof et al. (2019b) | **✓** | **✓** | **✓** | **NR** | **NR** | **NR** | **NR** | **✓** | **✓** | **✓** | Good |
| Vallof et al. (2020) | **✓** | **✓** | **NR** | **✓** | **✓** | **NA** | **✓** | **✓** | **✓** | **✓** | Good |

Symbols: ✓ - yes; X - no

Abbreviations: NR = not reported; NA = not applicable

**Table S2.** Risk of bias/quality assessment of the included studies using the NIH Quality Assessment Tool of Controlled Intervention Studies (Ma et al., 2020; NIH, 2013).

| **Study** | **Item** | | | | | | | | | | | | | | **Quality Rating** |
| --- | --- | --- | --- | --- | --- | --- | --- | --- | --- | --- | --- | --- | --- | --- | --- |
|  | **1** | **2** | **3** | **4** | **5** | **6** | **7** | **8** | **9** | **10** | **11** | **12** | **13** | **14** |  |
| Klausen et al. (2022) | **✓** | **✓** | **✓** | **✓** | **✓** | **✓** | **X** | **NR** | **NA** | **✓** | **✓** | **✓** | **✓** | **✓** | Good |

Symbols: ✓ - yes; X - no

Abbreviations: NR = not reported; NA = not applicable

**Table S3.** Risk of bias/quality assessment of the included studies using the NIH Quality Assessment Tool of Observational Cohort and Cross-Sectional Studies (Ma et al., 2020; NIH, 2013).

| **Study** | **Item** | | | | | | | | | | | | | | **Quality Rating** |
| --- | --- | --- | --- | --- | --- | --- | --- | --- | --- | --- | --- | --- | --- | --- | --- |
|  | **1** | **2** | **3** | **4** | **5** | **6** | **7** | **8** | **9** | **10** | **11** | **12** | **13** | **14** |  |
| Quddos et al. (2023) | **✓** | **✓** | **NA** | **✓** | **✓** | **✓** | **NA** | **NR** | **✓** | **X** | **✓** | **X** | **✓** | **✓** | Good |

Symbols: ✓ - yes; X - no

Abbreviations: NR = not reported; NA = not applicable

**Table S4.** Search Queries associated with GLP-1 and alcohol-consumption in preclinical, translation, and clinical studies

| **Search Queries** |
| --- |
| ("GLP-1" OR "Glucagon-Like Peptide-1" OR "Glucagon-Like Peptide 1" OR “GLP-1 Agonist” OR “Glucagon-Like Peptide-1 Agonist” OR “Glucagon-Like Peptide 1 Agonist” OR "Semaglutide" OR "Dulaglutide" OR “Trulicity” OR "Exenatide" OR “Liraglutide” OR “Lixisenatide” OR “Tirzepatide”) **AND** (“Alcohol” OR “Alcohol use disorder” OR “AUD” OR “Alcoholism” OR “Ethanol Administration” OR “Ethanol”) |
